# Biological sex-dependent differences in acute and post-acute stroke care—a population-based case–control study

**DOI:** 10.1093/esj/aakaf014

**Published:** 2026-01-01

**Authors:** Lukas Mayer-Suess, Kurt Moelgg, Heinrich Rinner, Christian Boehme, Anel Karisik, Benjamin Dejakum, Silvia Felicetti, Thomas Toell, Silvia Praxmarer, Johann Willeit, Stefan Kiechl, Michael Knoflach

**Affiliations:** Department of Neurology, Medical University Innsbruck, Innsbruck, Austria; Department of Neurology, Medical University Innsbruck, Innsbruck, Austria; Tyrolean Health Care Fund, Innsbruck, Austria; Department of Neurology, Medical University Innsbruck, Innsbruck, Austria; Department of Neurology, Medical University Innsbruck, Innsbruck, Austria; Department of Neurology, Medical University Innsbruck, Innsbruck, Austria; Department of Neurology, Medical University Innsbruck, Innsbruck, Austria; Department of Neurology, Medical University Innsbruck, Innsbruck, Austria; Tyrolean Health Care Fund, Innsbruck, Austria; Department of Neurology, Medical University Innsbruck, Innsbruck, Austria; Department of Neurology, Medical University Innsbruck, Innsbruck, Austria; VASCage, Research Center on Vascular Ageing and Stroke, Innsbruck, Austria; Department of Neurology, Medical University Innsbruck, Innsbruck, Austria; VASCage, Research Center on Vascular Ageing and Stroke, Innsbruck, Austria

**Keywords:** biological sex, ischemic stroke, outcome, population-based, stroke care pathway

## Abstract

**Introduction:**

Observed disparities in stroke care between the biological sexes are based on observational data from stroke centres or focus on single aspects of stroke care. Hence, we offer a comprehensive analysis encapsulating the entire stroke treatment path.

**Patients and methods:**

The quality-controlled, population-based Tyrolean Stroke Care pathway, recording all ischemic stroke cases in the entire federal state independent of treating hospital or department, was applied. Data from all patients (2019-2023) were analysed, which encompass information from stroke call activation to the time of recurrent stroke associated re-hospitalisation.

**Results:**

5733 ischemic stroke cases (men/women 56.0%/44.0%) were recorded with an incidence of first ever stroke of 133/100,000 inhabitants. Men were numerically more likely to suffer a stroke during that time period (149 vs 118/100,000 respectively). After adjusting for age, National Institute of Stroke Scale, and the pre-stroke modified Rankin Scale, no differences in pre-hospital stroke care, post-stroke rehabilitation access as well as most in-hospital metrics were seen. Still, women were less likely to be admitted to stroke units (odds ratio [OR] 0.89 [0.80, 1.00]) and less frequently underwent MRI (OR 0.85 [0.74, 0.96]) or echocardiography (OR 0.85 [0.76, 0.96]) during their hospital stay. However, women less frequently suffered serious post-stroke in-house complications (OR 0.80 [0.66, 0.97]). Upon follow-up, men had higher rates of all cause-mortality (OR 0.81 [0.69, 0.94]) as well as recurrent stroke-related re-admission (OR 0.63 [0.48, 0.83]).

**Conclusion:**

Within a highly structured and quality-controlled stroke care pathway, disparities in stroke care between sexes are low. Differences exist in terms of diagnostic algorithms, post-stroke mortality and recurrent stroke-related re-admissions, which merit further research.

## Graphical Abstract

**Graphical abstract:**
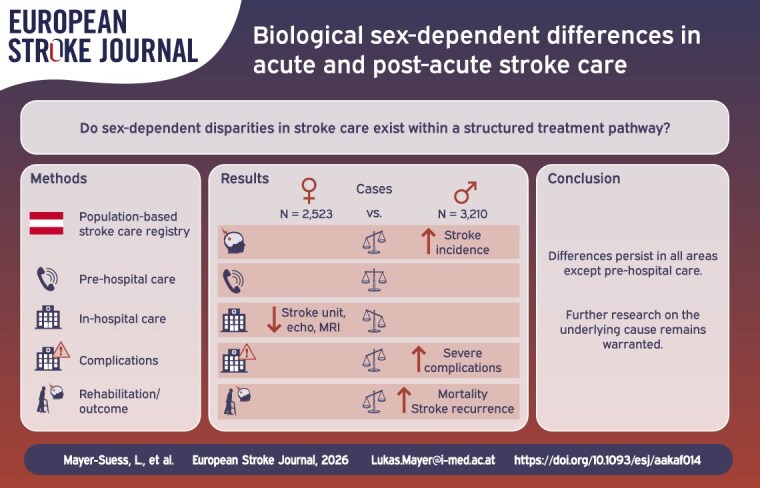

## Introduction

Ischemic stroke consistently ranks among the leading causes of mortality and long-term disability worldwide.^1^ This is true irrespective of the biological sex of stroke patients. Still, notable differences exist in the age distribution, prevalence of risk factors, clinical presentation and outcomes between the sexes.^2–5^ In addition to these clinical differences, disparities in stroke care, predominantly discriminating against women, have been found in numerous studies with a recent analysis confirming that women still remain underrepresented in clinical trials of endovascular treatment, intravenous thrombolysis and secondary prevention.^5^ This disparity is repeatedly attributed to the older age, higher rates of comorbidities and more severe neurological deficits in women suffering a stroke.^6^^,^^7^

Assessments of differences in stroke care between the sexes to date predominantly rely on single centre or regional analyses of mostly specialised stroke centres only. Seldomly, real-world population-based data is available and if so, only parts of the treatment path of individual stroke patients are presented. This entails a bias in addressing an important issue in the equity of stroke care between the sexes. Hence, our study aims to offer a comprehensive analysis encapsulating stroke incidence, care metrics, rehabilitation access, as well as recurrent stroke related re-hospitalisation (ie, an entire stroke care pathway) covering all hospital admissions with stroke for a whole federal region in the population based Tyrolean Stroke Care Pathway database.

## Patients and methods

For this study, we used the Tyrolean Stroke Care Pathway database, a population-based registry implemented in 2009 as a quality-of-care monitoring tool for ischemic stroke management.^8–11^ The database documents standardised stroke patient care from symptom onset to outpatient rehabilitation in the entire federal state of Tyrol. It is one of the few population-based registries worldwide, covering all ischemic stroke patients (ICD-10 Code i63 as the main diagnosis at discharge), independent of the treating hospital or department within the region with full data monitoring and no missing data. This is enabled by reimbursement-dependent mandatory data entry at discharge of ischemic stroke patients (ie, patients cannot be discharged from the treating hospital if data entry is not completed). We have previously shown that less than 2% of Tyrolean stroke patients are treated outside Tyrol and that 97.4% of stroke patients are indeed hospitalised, which implicates high data quality and completeness.^8^ Further, to limit false entry within the registry, codes I62, I64 and G46 are not permitted as the primary diagnosis in Tyrol, and tests of random samples of patients discharged with ICD-10 code G45 are performed by the data management team to ensure the data quality of differentiation between ischemic stroke and transitory ischemic attack patients.

### Patient recruitment and selection

All patients with a main residence in Tyrol, discharged from any of the 8 Tyrolean hospitals due to an acute ischemic stroke between 1 January 2019 and 31 December 2023, and therefore registered in the Tyrolean Stroke Care Pathway database, were included. Sex-dependent grouping was done according to biological sex. Study individuals were identified through the stroke discharge code I63.

### Variable definitions

All data collected from the entire patient treatment path from stroke call activation to re-hospitalisation within 1-year post index stroke were assessed. (1) Pre-hospital care encapsulates onset type (known, unknown, including wake-up stroke), type of primary transport to the initially treating hospital (private, ambulance, helicopter), rate of secondary hospital transfer, treating hospital type (comprehensive stroke centre or county hospital) as well as onset-to-door time. Further, to address potential confounders such as living situation and socioeconomic status, we included the degree of urbanisation as defined by Eurostat and insurance providers.^12^ Information on insurance providers enables an approximation of socioeconomic status as health insurance is mandatory in Austria, and is either covered by the BVAEB, which insures people working in the public sector (teachers, doctors etc.) and the SVS insuring self-employed persons (both considered a higher socioeconomic status) or the ÖGK, covering everyone else (considered less favourable socioeconomic status). (2) In-hospital care includes quality metrics of acute stroke management (door-to-imaging and door-to-needle-time), type of acute cerebral imaging performed (CT and/or MRI), rates of acute revascularisation measures (iv. thrombolysis and endovascular thrombectomy), as well as the rate of stroke unit care. Additionally, the frequency of receiving an MRI or an echocardiography (any type) was assessed, and the rate of early post-stroke rehabilitation measures as well as rehabilitation treatment types received were recorded (data on outpatient and rehabilitation centre admissions were available from 2021 to 2023). Further, the occurrence of post-stroke in-hospital complications was addressed. Complications were recorded individually and further dichotomised by severity, with severe complications defined as those necessitating intensive treatment resulting in a prolonged a prolonged in-hospital stay. Within our data set infections (pneumonia, sepsis), myocardial infarction, recurrent or progressive stroke, intracranial- or extracranial bleeding, epileptic seizure and deep vein thrombosis or pulmonary embolism are considered severe. (3) In the outpatient setting, overall rehabilitation access as well as type (rehabilitation centre or outpatient) is documented. In terms of outcome, we address the frequency of recurrent stroke-related re-hospitalisations 1-year after acute stroke admission and assess the time-to-recurrence. In an effort to enhance our outcome assessment, we include all-cause post-stroke mortality through data base linkage with our regional registry of death, managed and monitored by the Tiroler Landesstatistik.

### Statistical methodology

Statistical analyses were carried out using R 4.4.0, the dplyr (v1.1.4) and the gtsummary (v2.1.0) packages. Univariate differences between women and men were tested using the Wilcoxon rank sum test (for numeric variables), and Pearson’s Chi-squared test (for categorical variables). Adjusted odds ratios (ORs) (adjusting for age, or age, National Institute of Stroke Scale [NIHSS] and pre-stroke modified Rankin Scale [mRS]) were calculated by fitting logistic regression models. In case of non-dichotomous response variables, the first category is the reference level. Age-adjusted tests in case of numeric variables (log-transformations for onset-to-door-time, time to first neuroimaging, door-to-needle-time) were performed by fitting a multiple linear regression model. Kaplan–Meier curves for time to recurrent stroke were obtained with the survival (v3.8.3) and survminer (v0.5.0) packages. To assess time-to-recurrent ischemic stroke, cumulative incidence for competing risks data (recurrent stroke as the event of interest, death as the competing risk) was estimated with the tidycmprsk (v1.1.0) package.

### Standard protocol approvals, registration and patient consents

Analyses were approved by the local ethics committee at the Medical University of Innsbruck (EK#1283/2024). Data were collected as part of the governmental quality-assurance dataset of the Tyrolian Stroke Pathway based on the Tyrolean Healthcare Fund law (TGFG §18) and the federal law on health care documentation and target control health (Art. 15a Bundesverfassungsgesetz–Zielsteuerung Gesundheit). Therefore, individual patient consent was not required.

### Data availability

Study data that support the findings of this study are available from the corresponding author upon reasonable request after ethics approval and receipt of a signed data transfer agreement.

## Results

**Figure 1:**
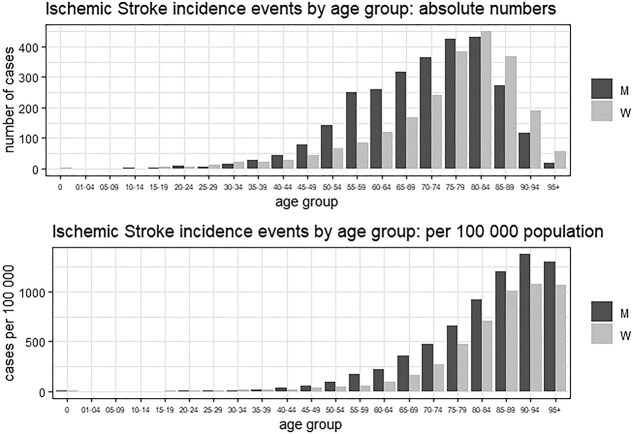
Difference in absolute number as well as incidence of first-ever ischemic stroke between both sexes dependent on age (per 100,000 inhabitants).

A total of 5733 ischemic stroke cases were recorded within our observation window (2019-2023). During this time, an overall population-based incidence of first ever stroke of 133 per 100,000 inhabitants was seen. Of special note, as one of the few regions worldwide, hospital admissions and quality parameters did not differ during the coronavirus disease (COVID) pandemic in Tyrol.^11^ Of the 5733 recorded stroke patients, 3210 (56.0%) were men and 2523 (44.0%) women. Conclusively and taking the population of Tyrol into account, the sex-specific incidence rate of first-ever stroke was numerically lower in women than in men (118 per 100,000 vs 149 per 100,000, respectively). Figure 1 presents the absolute number of ischemic stroke patients within age groups as well as the stroke incidence by age group. Herein, we present that, according to incidence rates, men more frequently suffered ischemic stroke in all age groups except between the ages of 25-34.

Table 1 presents the patient characteristics of our entire cohort, as well as those stratified by biological sex.

**Table 1:** Patient characteristics of all patients admitted due to acute ischemic stroke as well as group comparison dependent on biological sex

	**Entire cohort** ***n =* 5733**	**Men** ***n =* 3210**	**Women** ***n =* 2523**	***P*-value** ^**c**^	***P*-value** ^**d**^
Age^b^	76 (65, 83)	73 (63, 81)	79 (70, 85)	<.001	
Degree of urbanisation				.2	.7
Cities	940 (16.4)	528 (16.4)	412 (16.3)		
Towns and suburbs	2410 (42.0)	1320 (41.1)	1090 (43.2)		
Rural areas	2383 (41.6)	1362 (42.2)	1021 (40.5)		
Insurance providers				<.001	<.001
ÖGK	3851 (67.2)	2007 (62.5)	1884 (73.1)		
BVAEB/private	1882 (32.8)	1203 (37.5)	679 (36.9)		
mRS pre-stroke^a^				<.001	.004
0	3614 (63.0)	2139 (66.6)	1475 (58.5)		
1	811 (14.1)	450 (14.0)	361 (14.3)		
2	514 (9.0)	269 (8.4)	245 (9.7)		
3	457 (8.0)	215 (6.7)	242 (9.6)		
4	306 (5.3)	127 (4.0)	179 (7.1)		
5	31 (0.5)	10 (0.3)	21 (0.8)		
Pre-existing conditions^a^					
Diabetes	1191 (20.8)	724 (22.6)	467 (18.5)	<.001	<.001
History of ischemic stroke	1405 (24.5)	860 (26.8)	545 (21.6)	<.001	<.001
Atrial fibrillation	1620 (28.3)	829 (25.8)	791 (31.3)	<.001	.5
NIHSS^b^	3 (1, 7)	2 (1, 6)	3 (1, 9)	<.001	.5

^a^Values given as *N* (%).

^b^Values given as median (1st and 3rd quartile).

^c^Unadjusted *P*-values for differences between the biological sexes are given.

^d^Adjusted (age) *P*-values for differences between the biological sexes are given.

In unadjusted group comparison, women were older, had higher pre-stroke functional disability and higher NIHSS values and lower rates of pre-existing risk factors. The difference in risk factors (diabetes *P*^adj^ < .001, prior ischemic stroke *P*^adj^ < .001) as well as pre-stroke mRS (*P*^adj^ = .004) remained significant after adjusting for age at admission, while the difference in prior atrial fibrillation (*P*^adj^ = .5) and NIHSS at admission (*P*^adj^ = .5) disappeared upon this adjustment. There were no differences in living situation but women had a lower approximated socioeconomic status than men even after adjustment (*P*^adj^ < .001).

**Figure 2:**
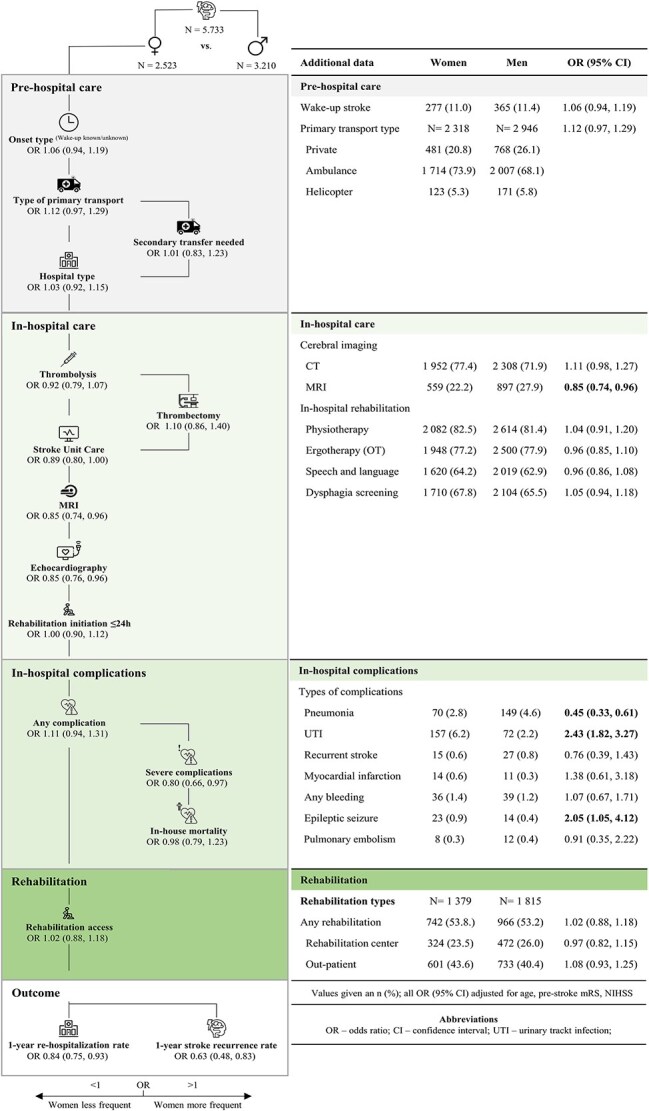
Differences in stroke care between the sexes within our population-based stroke care pathway.

Figure 2 depicts the sex-specific differences in pre-hospital and in-hospital care, rehabilitation access, as well as outcomes. In brief, after adjusting for age, stroke severity (NIHSS) and pre-stroke mRS, women did not significantly differ in pre-hospital care to men. In addition to these findings, onset-to-door time did not differ between the sexes (women vs men in minutes [IQR]—119 [60, 315] vs 120 [60, 335]; OR 1.09 [0.98, 1.23]).

During the in-hospital stay, quality metrics of acute stroke care, such as door-to-imaging- (women vs men in minutes [IQR]—25 [16, 54] vs 26 [16, 59]; OR 0.99, [0.88, 1.11]) and door-to-needle-time (women vs men in minutes [IQR]—41 [28, 62] vs 43 [28, 63]; *P* = 0.9) as well as rate of acute revascularisation measures did not differ between men and women (men vs women—iv thrombolysis 18.0% vs 17.3%; OR 0.92 [0.79, 1.07]; thrombectomy 5.3% vs 5.6%; OR 1.10 [0.86, 1.40]). However, women were (i) slightly less likely to be admitted to stroke units (men vs women—63.7% vs 59.2%; OR 0.89 [0.80, 1.00]) and (ii) less likely to undergo MRI imaging (men vs women—27.9% vs 22.2%; OR 0.85 [0.74, 0.96]) or echocardiography (men vs women—71.4% vs 63.3%; OR 0.85 [0.76, 0.96]) as diagnostic tools during their hospital stay. These differences did not disappear when adding atrial fibrillation to the confounding variables. In-hospital complications differed between the sexes, as women were more prone to urinary tract infections (UTI—men vs women—2.2% vs 6.2%; OR 2.43 [1.82, 3.27]) as well as epileptic seizures (men vs women—0.4% vs 0.9%; OR 2.05 [1.05, 4.12]), while men more frequently had pneumonia (men vs women—4.6% vs 2.8%; OR 0.45 [0.33, 0.61]). Of note however, women less frequently experienced severe complications (OR 0.80 [0.66, 0.97]), which did not relate to a difference in in-house mortality rate between the sexes (men vs women—5.9% vs 8.5%; OR 0.98 [0.79, 1.23]).

**Figure 3:**
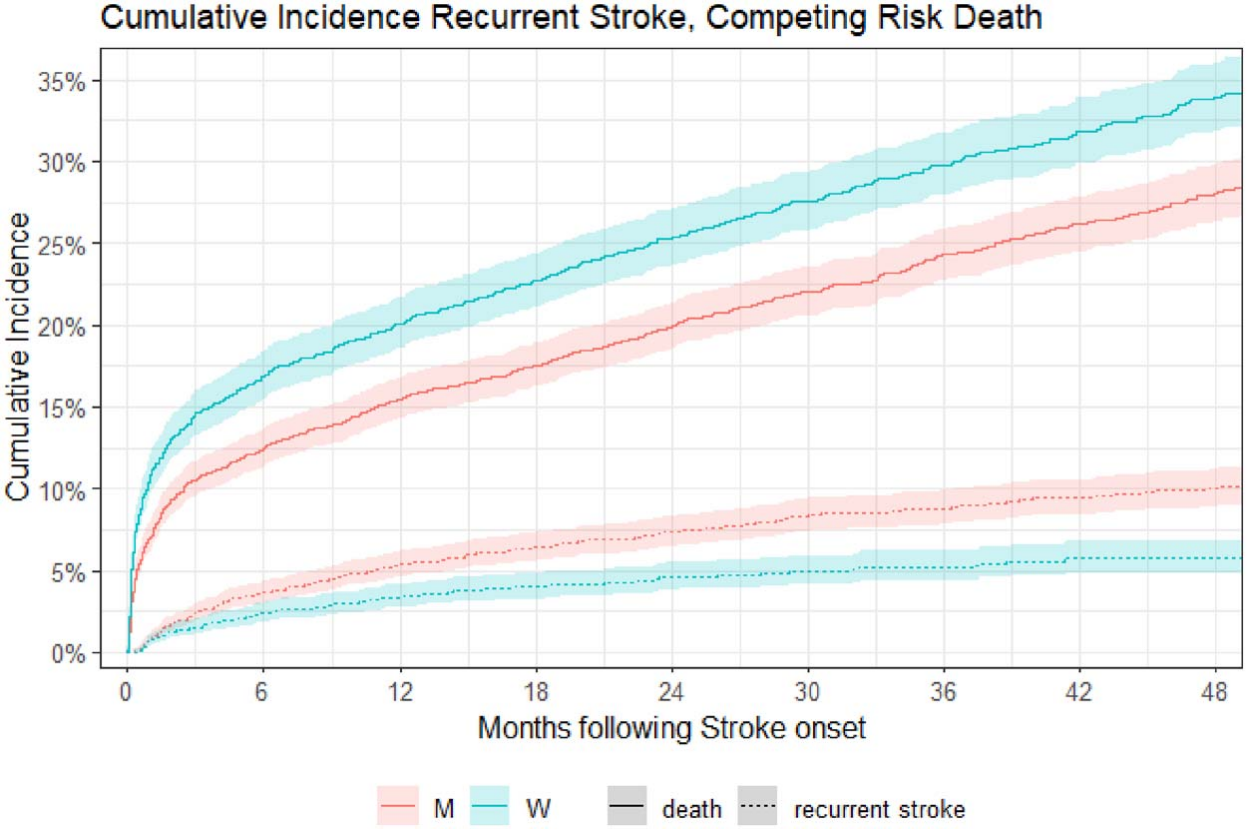
Increased unadjusted 1-year mortality post-stroke in women with re-admissions due to recurrent stroke remaining higher in men than in women within a competing risk model.

No difference was seen concerning post-stroke rehabilitation access between the sexes. Upon follow-up, women had higher rates of all-cause mortality 1-year post-stroke (men vs women—16.6% vs 20.8%), but this disappears when adjusting for age and even reverses when adjusting for age, NIHSS and mRS (OR 0.81 [0.69, 0.94]). Men were further more likely to have a recurrent stroke-related re-admission (OR 0.63 [0.48, 0.83]), even if including all-cause mortality in a competing risk model (Figure 3).

## Discussion

We present a comprehensive population-based assessment of biological sex-dependent differences in stroke care along an entire treatment path from stroke call activation, to in-hospital management and post-stroke rehabilitation to the frequency of re-hospitalisation within the first year. Through our assessment of 5733 consecutive ischemic stroke patients treated in Tyrol, we report the following key results.

In line with literature, women in general had a lower incidence of ischemic stroke in Tyrol than men.^13–16^ Attributable factors for the lower stroke incidence in women, especially in younger age groups, have been discussed in literature for many years. Some accredit the higher frequency and earlier onset of traditional risk factors in men, some the protective effect of oestrogen in women but the difference may also reflect a discrepancy in itself as women are known to be more frequently discharged with stroke mimics as their key diagnosis.^17–21^

No difference in pre-hospital stroke care can be reported, which, in most parts extended to in-hospital and post-stroke care (Figure 2). It is especially promising that quality metrics of acute stroke management, such as onset-to-door-, door-to-imaging- and door-to-needle times as well as rates of iv thrombolysis or endovascular thrombectomy, did not differ between the sexes. This finding, as well as the equivalent access to in-hospital and post-discharge rehabilitation for men and women, extends on previous analyses from the Tyrolean Stroke Care Pathway database.^10^^,^^11^ Our data stands in contrast to other studies that show pronounced differences in the quality of stroke care.^21–25^ It is very tempting to speculate, that this is largely attributable to the positive impact of a structured treatment pathway limiting the potential of inherent biases affecting individual treatment decisions. The quality-controlled Tyrolean Stroke Care Pathway has been in place for more than 15 years and has shown to profoundly improve stroke care and stroke outcomes and was resilient to the impact of COVID-19 associated restrictions.^8^^,^^11^

However, even in our setting, slight differences between the sexes have to be reported. Women were less likely to be admitted to a stroke unit and during in-hospital etiologic workup less frequently received MRI and echocardiography (Figure 2). All 3 of these aspects are considered standard in high quality stroke care. These disparities have previously been observed in other studies, but were primarily attributed to sex differences in patient age, stroke severity and/or pre-stroke mRS. In our population-based data adjusting for such confounders did not alter the result.^26–31^ An incomplete stroke workup could contribute to the observation in literature, that women are more frequently diagnosed with stroke mimics compared to men even if experiencing the same symptoms, which may put our finding of lower stroke incidence in women in perspective.^32^ Concerning the individual differences seen within our dataset, it may well be that systemic biases have an impact on clinical decision making, which in turn results in less than optimal stroke care in specific patient groups. Such biases may stem from the dissimilarity in clinical presentation or communication of patients subjective symptoms between the sexes.^33^^,^^34^ In the Tyrolean Stroke Care pathways case, however, such systemic biases should be limited as patient care is structured and quality controlled. Still, this pathway does not encapsulate detailed information on specific gender-related patient characteristics that may have an influence on pre-hospital as well as hospital care and are worth exploring, such as living situation, caregiver status or socioeconomic status.

Differences in post-stroke complications within our analysis exist between the sexes with women exhibiting lower rates of hospital-acquired pneumonia and higher rates of UTI in the in-hospital setting compared to men (Figure 2). This is in line with previous studies, even if the pneumonia rate within our study was better than in a recently published meta-analysis.^35–40^ Mechanistic explanations for these differences in post-stroke infections include anatomic situs, clinical presentation (ie, frequency of post-stroke dysphagia) as well as variation in sex hormones.^21^^,^^41^^,^^42^ Still, a definitive reason has yet to be established. Even though severe post-stroke complications, defined as those necessitating intensive treatment and resulting in a prolonged in-hospital stay, were more common in men, they did not translate to an increase of in-hospital mortality. Upon outpatient care however, and after adjusting for age, NIHSS and pre-stroke mRS, men had higher rates of all-cause mortality post-stroke (OR 0.81 [0.69, 0.94]; *P* = 0.006) and were more frequently re-hospitalised within 1-year after stroke due to stroke recurrence (Figure 3). The re-admission rate remained higher in men even when calculating a competing risk model including all-cause post-stroke mortality (Figure 3). There have been reports delivering mixed results on re-hospitalisation rates in previous studies.^43^^,^^44^ One contributor might be that women are more commonly discharged to nursing homes, where re-hospitalisation might be less necessary than in patients in informal home-care.^45^^,^^46^ Within a limited dataset, consisting of those patients not being admitted to a stroke unit (40% of the complete cohort), 15% of women and 3.6% of men were discharged to nursing homes. Considering the overall distribution of nursing home residents being 70% female and 30% male, according to the federal nursing department of Tyrol, it is tempting to believe that discharge setting is a key contributor to our re-hospitalisation finding. As the results are however rather speculative and derived from a limited dataset, this explanation has to be interpreted with caution, especially as literature proclaims a tendency towards potentially avoidable hospitalisations being increased over the last decade in residents of long-term care facilities.^47^^,^^48^

Even though our population-based approach assessing differences in stroke care between the sexes is based on high-quality and complete data, we have to acknowledge the following limitations that have to be considered when interpreting our findings: The Tyrolean Stroke Care pathway is one of the few population-based stroke registries encompassing the entire treatment path of ischemic stroke patients. It was designed as a quality control instrument to detect regional disparities in stroke care in Tyrol. Due to its population-based mandatory nature, the list of variables collected is kept as low as possible and reliably and reproducibly deducted from clinical routine practice and assessments. Even though we adjusted for stroke severity and pre-stroke disability, we do not have detailed information on comorbidities, risk factors (as well as control), general frailty, socioeconomic status and living situation that might confound our data. These missing data in part limit our results validity on gender-related aspects of stroke care, which may also have an impact on stroke workup, stroke unit care and re-hospitalisation rates. Additionally, the Tyrolean Stroke Care Pathway database does not routinely collect data on functional- or patient-reported outcomes. Further, the database does not record discharge settings for all patients or emergency department visits upon follow-up, which may also be a confounding factor to recurrent stroke related re-hospitalisations. Lastly, we acknowledge that individual items addressed within our analysis touch on gender- rather than biological sex-dependent differences in clinical care (eg, socioeconomic status, living situation).

In summary, within a highly structured and quality-controlled stroke care pathway, disparities in stroke care between sexes are low. Collectively, the lower odds of women being admitted to a stroke unit and receiving MR-imaging or echocardiography during hospitalisation as well as the higher re-hospitalisation rates of men merits further research.

## Figures and Tables

**Table:** 

**Name**	**Affiliation**	**Role**	**Contribution**
Theresa Geley, MD	Tyrolean Health Care Fund, Innsbruck, Austria.	Study group member	Data collection
Johannes Schöch, MD	Tyrolean Health Care Fund, Innsbruck, Austria.	Study group member	Data collection
Ivo Grazaidei, MD	Hospital Hall in Tirol, Hall, Austria.	Study group member	Data collection
Thomas Erlacher, MD	Hospital Hall in Tirol, Hall, Austria.	Study group member	Data collection
Andreas Maurer	Emergency Medical Service, Innsbruck, Austria.	Study group member	Data collection
Adolf Schinnerl, MD	Emergency Medical Service, Innsbruck, Austria.	Study group member	Data collection
Alexandra Rauter-Rzehak, MD	Hospital Schwaz, Schwaz, Austria.	Study group member	Data collection
Hannes Gänzer, MD	Hospital Schwaz, Schwaz, Austria.	Study group member	Data collection
Elke Pucks-Faes, MD	Hospital Hochzirl, Zirl, Austria.	Study group member	Data collection
Heinrich Matzak, MD	Hospital Hochzirl, Zirl, Austria.	Study group member	Data collection
Klaus Engelhardt, MD	Doctors in private practice, Tyrol, Austria.	Study group member	Data collection
Heinrich Spiss, MD	Doctors in private practice, Tyrol, Austria.	Study group member	Data collection
Artur Mair, MD	Doctors in private practice, Tyrol, Austria.	Study group member	Data collection
Patrick Loidl, MD	Hospital Reutte, Reutte, Austria.	Study group member	Data collection
Gudrun Schoenherr, MSc	Medical University Innsbruck, Innsbruck, Austria.	Study group member	Data collection
Karin Willeit, MD, PhD	Medical University Innsbruck, Innsbruck, Austria.	Study group member	Data collection
Raimund Pechlaner, MD, PhD	Medical University Innsbruck, Innsbruck, Austria.	Study group member	Data collection
Eva Hametner, MD	Medical University Innsbruck, Innsbruck, Austria.	Study group member	Data collection
Christoph Schmidauer, MD	Medical University Innsbruck, Innsbruck, Austria.	Study group member	Data collection
Martin Sojer, MD	Medical University Innsbruck, Innsbruck, Austria.	Study group member	Data collection
Christian Brenneis, MD	Rehabilitation Center Münster, Münster, Austria.	Study group member	Data collection
Josef Grossmann, MD	Hospital Lienz, Lienz, Austria.	Study group member	Data collection
Matthias DalBianco, MD	Hospital Lienz, Lienz, Austria.	Study group member	Data collection
Markus Mayr, MD	Hospital Kufstein, Kufstein, Austria	Study group member	Data collection
Klaus Seppi, MD	Hospital Kufstein, Kufstein, Austria	Study group member	Data collection
Armin Krösbacher, MD	Dpt. Of Anesthesiology, Medical University Innsbruck, Innsbruck, Austria.	Study group member	Data collection
Peter Rainer, MD	Hospital St Johann in Tirol, St Johann in Tirol, Austria.	Study group member	Data collection
Ewald Wöll, MD	Hospital Zams, Zams, Austria.	Study group member	Data collection
Bernhard Oberwinkler, MD	Austrian Health Insurances	Study group member	Data collection
Bernhard Huter	Austrian Health Insurances	Study group member	Data collection
Andreas Tür, MSc	Innsbruck University Hospital	Study group member	Data collection
Julia Runge, MA	Innsbruck University Hospital	Study group member	Data collection

## Trial registration

Not applicable due to the study not being a clinical trial.
